# Cognitive effects of dopaminergic treatment in Alzheimer's disease: Systematic review and meta‐analysis

**DOI:** 10.1002/trc2.70142

**Published:** 2025-08-20

**Authors:** Cristina Bonet Olivares, Michael C. B. David, Marta Estrada Obeso, Martina Del Giovane, Suzanne Reeves, Paresh A. Malhotra

**Affiliations:** ^1^ UK Dementia Research Institute Care Research and Technology Centre White City Campus Imperial College London and the University of Surrey London UK; ^2^ Department of Brain Sciences Charing Cross Campus Imperial College London UK; ^3^ Division of Psychiatry University College London London UK

**Keywords:** Alzheimer's disease, cognitive symptoms, dopaminergic system, meta‐analysis, monoamine oxidase B inhibitors, noradrenergic, pharmacological interventions, systematic review

## Abstract

**INTRODUCTION:**

Despite advances in disease‐modifying drugs, better treatments for symptomatic Alzheimer's disease (AD) are needed, with dopaminergic neurotransmission representing a potential target. The objective of this systematic review and meta‐analysis was to evaluate the efficacy of drugs with predominantly dopaminergic action in improving cognitive symptoms in AD.

**METHODS:**

The MEDLINE, Embase, and ClinicalTrials.gov databases were searched from 1980 to January 2023. We used random effect models to generate pooled effect estimates

**RESULTS:**

We included 19 prospective randomized controlled AD trials (1408 total participants), of which 7 were of “good” quality, 8 “fair,” and 4 “poor.” All were included in the analysis. The overall pooled effect was small but showed a significant positive effect of dopaminergic drugs compared to placebo (standardized mean difference [SMD]: 0.33, 95% confidence interval [CI]: 0.08 to 0.59, *P* = 0.01; *I* ^2^ = 79%). Significance remained after removing outliers to account for heterogeneity. When exploring subgroups (divided by mechanism of action), 5 trials of dopamine reuptake inhibitors did not show a significant effect on cognition, whereas 12 monoamine oxidase B (MAO‐B) inhibitor trials showed a moderately significant positive effect (SMD: 0.52, 95% CI: 0.13 to 0.90, *P* = 0.01; *I* ^2^ = 84%).

**DISCUSSION:**

We show evidence of the benefit of dopaminergic medications, specifically MAO‐B inhibitors, on cognitive symptoms in AD. Several studies included here also used drugs with both noradrenergic and dopaminergic action, highlighting a potential dual stimulation that could lead to better clinical efficacy. Trials targeting well‐defined patient populations, ideally supported by biomarker evidence of dopaminergic dysfunction, are needed to compare noradrenergic and dopaminergic agents—both separately and in combination—on cognitive function to maximize treatment effects. Particularly, further research should explore the impact of MAO‐B drugs on specific aspects of cognitive function to better understand their mechanism given the upregulation of MAO‐B expression in AD.

**Highlights:**

- We conducted a meta‐analysis investigating the efficacy of dopaminergic drugs in improving cognitive symptoms in Alzheimer's disease (AD).
- Our findings highlight the potential cognitive benefits of dopaminergic medications, particularly monoamine oxidase B inhibitors, in AD.
- Future trials are warranted and could focus on biomarker‐defined patient groups to enhance effectiveness.

## 1 INTRODUCTION

Even with the advent of potentially disease‐modifying drugs, there is a clear need to improve the current treatments for Alzheimer's disease (AD). Largely overlooked as a site of pathology in AD, the substantia nigra (SN)–dopaminergic system represents a potential target.

Lewy body, tau aggregation, and neuronal loss occur in the SN in AD.^1^, ^2^, ^3^ Reduced endogenous dopamine levels and disruption of mesocorticolimbic dopaminergic neurotransmission in AD have been demonstrated through positron emission tomography (PET) imaging and *post mortem* studies, alongside meta‐analysis evidence showing decreases in dopamine and associated receptors.^4^, ^5^, ^6^ In an AD mouse model, apomorphine, a dopaminergic drug, has been shown to reduce amyloid beta (Aβ) and tau pathology, improving cognition.^7^ Further, increasing cortical dopamine, via reuptake blocker^8^ or levodopa,^9^ attenuates memory impairment in mouse models.

There are multiple potential mechanisms for drugs to boost dopaminergic transmission. Included here, lisuride^10^ and rotigotine^10^ are dopamine receptor agonists. Selegiline and rasagiline inhibit monoamine oxidase B (MAO‐B)—a key enzyme in dopamine breakdown^11^, ^12^ Ladostigil similarly inhibits MAO‐B but interestingly also acetylcholinesterase.^13^

In the 1990s, a series of small, likely underpowered, clinical trials tested a range of dopaminergic drugs in AD. Additionally, interest has grown in the potential of drugs with joint noradrenergic/dopaminergic action.^14^ Given that, to our knowledge, these trial results have not previously been aggregated, and considering the pathophysiological evidence, we performed a systematic review and meta‐analysis following Preferred Reporting Items for Systematic Reviews and Meta‐Analyses (PRISMA) guidelines (Table S1 in supporting information) of predominantly dopaminergic drugs in AD, evaluating evidence for benefit on cognition. While included compounds are not “pure” in their mechanism of action, inclusion was based on the likely primary mechanism at the doses used (including methylphenidate and bupropion, deemed to have sufficient action on both noradrenergic and dopaminergic systems).^15^, ^16^

## 2 METHODS

We searched MEDLINE, Embase, and ClinicalTrials.gov (1980 to January 2023) and manually searched further sources, including reviews and conference abstracts, for trials fulfilling the following criteria: (1) study populations defined as patients with AD or mild cognitive impairment, (2) prospective randomized controlled trials comparing dopamine‐enhancing drugs or dopamine receptor agents versus placebo, (3) cognitive outcomes. Single‐dose studies and those targeting multiple neurotransmitter systems without evidence of predominant dopaminergic action were excluded. Full criteria are in Table S2 in supporting information. The cognitive measure used was the Mini‐Mental State Examination (MMSE; where available), otherwise the Alzheimer's Disease Assessment Scale—Cognitive subscale (ADAS‐Cog), or memory‐specific measures (Table 1). Two reviewers (M.C.B.D., M.E.O.) independently screened titles and abstracts and discussed eligibility with arbitration by P.M. (Figure S1 in supporting information). Study quality was assessed using the National Heart, Lung, and Blood Institute Quality Assessment of Systematic Reviews and Meta Analyses.^17^

**TABLE 1:** Table detailing the nature of all studies included in the meta‐analysis.

Included dopaminergic Alzheimer's disease studies									
Study	Participant demographics				Intervention				Outcome measure
	Mean age (years)	% Female	*N*		Class	Drug	Daily dosage (mg)	Duration (weeks)	
			Drug	Placebo					
Herrmmann (2008)^a^, ^18^	77.9	53.8	13	12	DRI	Methylphenidate	20	2	MMSE
Lanctôt (2014)^19^	76.0	61.6	29	31	DRI	Methylphenidate	20	6	MMSE
Maier (2020)^20^	74.8	38	54	54	DRI	Bupropion	150–300	12	MMSE
Mintzer (2021)^21^	76.0	34	99	101	DRI	Methylphenidate	20	26	MMSE
Padala (2018)^22^	77.0	0	30	30	DRI	Methylphenidate	10–20	12	Modified MMSE
Claus (1998)^23^	74.1	50	10	12	DR agonist	Lisuride	0.3	12	MMSE
Filip (1999)^24^	83.0	71	91	51	MAO‐B inhibitor	Selegiline	10	24	MMSE (Orientation)
Koch (2020)^25^	73.9	62	47	47	DR agonist	Rotigotine	2–4	24	ADAS‐Cog
Matthews (2021)^26^	74.0	50	25	25	MAO‐B inhibitor	Rasagiline	0.5–1	24	MMSE
Tariot (1998)^27^	69.9	64	25	24	MAO‐B inhibitor	Selegiline	10	16	MMSE‐18
Schneider (2002)^1^	71.4	37.6	99	103	MAO‐B inhibitor	Ladostigil	10	156	MMSE
Sano (1996)^28^	73.4	65	115	96	MAO‐B inhibitor	Selegiline	10	108	MMSE
Mangoni (1991)^29^	68.8	64	62	46	MAO‐B inhibitor	Selegiline	10	13	Wechsler Memory Scale
Lawlor (1997)^30^	75.0	70	5	6	MAO‐B inhibitor	Selegiline	10	12	ADAS‐Cog
Freedman (1996) ^31^	70.4	53	21	24	MAO‐B inhibitor	Selegiline	10	25	MMSE
Finali (1991)^32^	62.5	42	9	10	MAO‐B inhibitor	Selegiline	10	26	Rey Auditory‐Verbal Learning Test
Burke (1993)^33^	73.1	61	17	15	MAO‐B inhibitor	Selegiline	10	65	MMSE
Agnoli (1992)^34^	68.6	60	5	5	MAO‐B inhibitor	Selegiline	10	9	Randt Memory Index
Agnoli (1990)^35^	70.4	40	9	9	MAO‐B inhibitor	Selegiline	10	13	Randt Memory Index
Mean/total over all studies:	74.2	50.5	765	701	N/A	N/A	N/A	30.8	N/A

Abbreviations: ADAS‐Cog, Alzheimer's Disease Assessment Scale‐Cognitive subscale; DR, dopamine receptor; DRI, dopamine reuptake inhibitor; MAO‐B, monoamine oxidase B; MMSE, Mini‐Mental State Examination.

^a^ Indicates cross‐over design.

Meta‐analysis was conducted using Review Manager V5.4.^36^ Changes in group means from baseline to final timepoint were calculated for both groups. For outcome measures where negative change in score indicated improvement, scores were multiplied by −1. As all outcomes were continuous, and different measures were used, standardized mean differences (SMDs) with 95% confidence intervals (CIs) were calculated using an inverse variance random effects model. Standard deviations were imputed where not reported.^37^ As heterogeneity could influence outcomes, we used random effects meta‐analysis models to estimate SMDs. Heterogeneity was measured using the *I* ^2^ statistic. Funnel plots were created using JASP^38^ to represent effect sizes and identify publication bias, quantified with Egger tests.^39^ Studies were considered outliers if their 95% CIs did not overlap with the pooled effect's intervals.

Post hoc meta‐regression analyses assessed whether age, sex, treatment duration, and publication year affected results for global cognition and apathy. Each covariate was analyzed separately, reporting the number of studies, covariate estimate (ß), *P* value, and proportion of variance (*R* ^2^).

## 3 RESULTS

Table 1 shows the baseline characteristics of included trials. There were 19 trials, with treatment duration between 2 and 156 weeks. Four trials included in our previous meta‐analysis involving drugs with joint noradrenergic/dopaminergic action were included here.^40^ Participant numbers ranged from 10 to 211 (total = 1408), with the mean age from 62.5 to 83.0 years. The most common drugs were MAO‐B inhibitors (12 studies), followed by dopamine reuptake inhibitors (5 studies) and dopamine receptor agonists (2 studies). Seven studies were of “good” quality, eight were “fair,” and four were “poor” (Table S3 in supporting information); all were included.

**RESEARCH IN CONTEXT**

1. **Systematic review**: We searched for randomized controlled trials investigating the efficacy of drugs with predominately dopaminergic action on cognitive symptoms of Alzheimer's disease (AD). We searched MEDLINE, Embase, and ClincalTrials.gov from 1980 to 2023. Meta‐analysis was conducted to generate estimates of pooled size effects.
2. **Interpretation**: Based on data from 19 studies with a total of 1408 participants, we found a small but significant positive effect of these drugs on cognition compared to placebo. Monoamine oxidase‐B (MAO‐B) inhibitors in particular showed a moderate significant positive effect. There was significant heterogeneity. Dopaminergic dysfunction may represent an under‐recognized and treatable component of AD pathophysiology.
3. **Future directions**: Our findings provide justification for further evaluation of dopaminergic treatments in AD. Studies could incorporate biomarkers to quantify dopaminergic dysfunction at the individual level, explore combination therapies with other neurotransmitter‐modulating drugs, and analyze efficacy across both neuropsychiatric and cognitive outcomes.

The overall pooled effect size showed a small^41^ but significant positive effect of dopaminergic drugs compared to placebo (SMD: 0.33, 95% CI: 0.08 to 0.59, *P* = 0.01; *I* ^2^ = 79%). The Egger test was not significant (*P* > 0.05; Figure S2 in supporting information). After removing the outlier (Mangoni et al.^29^), the effect remained significant, and heterogeneity was reduced but remained significant (*P* = 0.02). Removal of the “poor”‐quality studies conferred almost no change (SMD: 0.32, 95% CI: 0.01 to 0.63, *P* = 0.04; *I* ^2^ = 83%).

**FIGURE 1:**
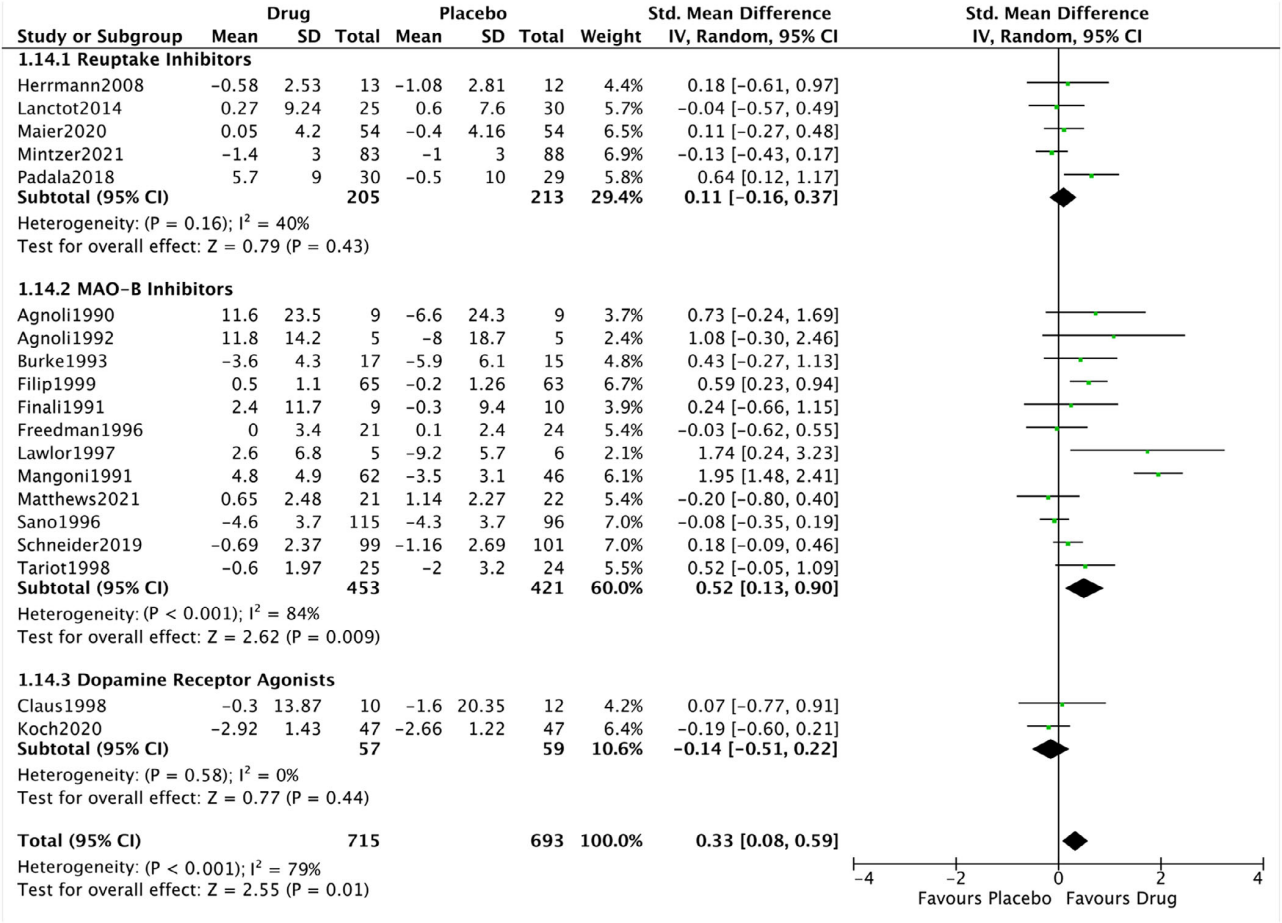
Forest plot of dopaminergic drugs on cognition, split by class. Comparison of drug and placebo for effect on measures of cognition between baseline and end of treatment. CI, confidence interval; IV, inverse variance; MAO‐B, monoamine oxidase B; SD, standard deviation.

Studies were split into subgroups based on class of action; dopamine reuptake inhibitors, MAO‐B inhibitors, and dopamine receptor agonists. The first two sub‐groups were large enough to analyze separately. The five trials of dopamine reuptake inhibitors showed no significant effect on cognition (Figure 1). However, the 12 MAO‐B inhibitor studies showed a moderate^41^ positive effect versus placebo (SMD: 0.52, 95% CI: 0.13 to 0.90, *P* = 0.01; *I* ^2^ = 84%), with considerable heterogeneity (Figure 1). We also conducted a sensitivity analysis splitting the drugs into those with a dual noradrenergic–dopaminergic action (reuptake inhibitors) and those with purer dopaminergic action (receptor agonists and MAO‐B inhibitors). The former did not show a significant effect (SMD: 0.11, 95% CI: −0.16 to 0.37, *P* = 0.43; *I* ^2^ = 40%). However, the latter did (SMD: 0.42, 95% CI: 0.08 to 0.77, *P* = 0.02; *I* ^2^ = 93%).

We then performed a sensitivity analysis in which we split trials based on those that used global cognition as their outcome measure and those that used memory‐specific tests. The trials that had global cognition, which were all except Agnoli et al ^34^, Agnoli et al ^35^, Mangnoi et al ^29^, and Finali et al ^32^, gave a non‐significant result (SMD: 0.15, 95% CI: −0.02 to 0.32, *P* = 0.09; *I* ^2^ = 48%). On the other hand, the trials that used memory as their outcome measure gave a significant result in favor of drug (SMD: 1.05, 95% CI: 0.12 to 1.97, *P* = 0.03, *I* ^2^ = 78%), meaning greater improvement in the drug group than in controls.

Meta‐regression showed a significant effect of year of publication (ß = −0.02, 95% CI: −0.04 to 0.00, *P* = 0.02), suggesting that the older studies had a greater effect size. Other covariates were not significant (Table S4 and Figure S3 in supporting information).

## 4 DISCUSSION

This systematic review and meta‐analysis found mixed quality evidence that dopaminergic pharmacotherapies improve cognition in AD.

A spectrum of dopaminergic insufficiency likely exists in AD, influencing therapeutic efficacy.^42^ For those with normal dopaminergic tone, exogenous enhancement could induce side effects, notably motor and psychiatric ones. In the studies reviewed here, side effects were generally uncommon, with isolated reports of psychiatric symptoms like hallucinations or agitation. However, given the experience with dopaminergic agents in other clinical populations, such as Parkinson's disease (PD), careful monitoring remains critical in future trials. For example, MAO‐B inhibitors, like selegeline, are associated with side effects like sleep disturbances, anxiety, nausea, and hallucinations.^43^ Therefore, even with evidence of group‐level efficacy and safety, risk–benefit analysis at the individual level is necessary. Dopaminergic system biomarkers to direct and monitor treatment could be considered to aid this practice. Neuromelanin‐sensitive magnetic resonance imaging and pupillometry^44^ have potential utility for this purpose, along with Ioflupane single‐photon emission computed tomography imaging, an established diagnostic tool in PD that provides a functional index of the dopaminergic system. However, it should be noted that dopaminergic drugs might still provide cognitive benefits even in individuals without significant deficits. While the extent of dopaminergic dysfunction in AD may be variable, these drugs are well known to enhance cognition in healthy people.^45^ Because individuals with AD have a lower cognitive baseline, they may experience more benefit compared to cognitively normal individuals, even if the drugs are not correcting a neurochemical deficit per se.^45^

We included compounds with noradrenergic and dopaminergic action, such as methylphenidate, as dual stimulation might have greater clinical efficacy than singular approaches.^14^ However, sub‐group analysis of dopaminergic drug types, dividing into dopamine/noradrenaline reuptake inhibitors and MAO‐B inhibitors, only showed significant effects for the latter. A fully powered comparison between selective noradrenergic and dopaminergic agents and single drugs with combined action, such as methylphenidate, is needed. Regarding MAO‐B inhibitors, the 2003 Cochrane Review and meta‐analysis of selegeline concluded there was no evidence of a clinically meaningful benefit. However, the authors noted cognitive improvements compared to placebo at 4 to 6 weeks and 8 to 17 weeks, just not over longer time scales, and the positive results were dismissed due to study heterogeneity.^46^ Four trials in that meta‐analysis were not included here, as the data were inaccessible or did not meet our criteria. All four showed positive effects, although only one was significant. Additionally, given the evidence from PET showing a region‐specific pattern of MAO‐B up‐regulation in AD, there is a good rationale for the potential efficacy of this class of drug.^47^ Notably, trials using memory‐specific outcomes, rather than global cognition, showed a significant effect. This is a potentially important distinction given that memory impairment is the primary cognitive deficit in AD. Together with our results, further well‐powered, long‐term trials are needed to clarify the therapeutic potential of dopaminergic modulation in AD. Head‐to‐head comparisons of dual dopamine/noradrenaline agents versus dopamine‐selective agents could reveal whether combined mechanisms offer a greater clinical benefit.

Our study had limitations. Drug inclusion criteria were based on a threshold for dopaminergic action, not fixed pharmacokinetic/pharmacodynamic metrics. The treatments included have varying mechanisms and activity on other systems, reflective of the fact that clinically used drugs rarely exhibit total pharmacological specificity. Importantly, the common effect of all included compounds lies in their shared dopaminergic action. Also, different outcome tools were used across studies. Most studies reported mean score change at the group level, whereas individual‐level changes may have provided more accurate results. Finally, we did not model baseline performance or symptom severity, which may account for some variation in treatment response. Furthermore, the use of global cognition as an outcome measure limited interpretation, as we were unable to establish the impact of interventions on specific aspects of cognitive function, which may be more sensitive to change. For example, the use of D2/3 dopamine receptor agonists as an adjunctive treatment in AD may differently affect cognition and motor function, potentially increasing processing speed at the expense of attentional function.^48^ The quality of studies included was mixed, with 7 of the 19 rated as “good.”

However, given that the key strength of a meta‐analysis lies in its ability to integrate evidence across studies of varying quality, we decided to retain all the studies to preserve the power of our analysis. We acknowledge that such studies may introduce bias, limiting the precision of our analysis. Thus, the conclusions drawn should be interpreted with some caution given that there was considerable remaining heterogeneity across studies (*I* ^2^ of 44%) even after removing the outlier. While other factors such as age, treatment duration, and sex were examined via meta‐regression, other potential sources of heterogeneity such as baseline cognitive scores and drug doses were not assessed. Future high‐quality studies are needed to further strengthen the evidence. Additionally, the meta‐regression revealed that older studies had a disproportionately large effect size, which suggests potential bias.

To conclude, we present evidence for the efficacy of dopaminergic medications, particularly MAO‐B inhibitors, for cognitive symptoms in AD. This highlights their potential for targeted use in specified patient subgroups and suggests further, well‐powered trials of such treatments, using sensitive outcome measures, are justified.

## CONFLICT OF INTEREST STATEMENT

The authors declare no conflicts of interest. Author disclosures are available in the supporting information.

## Supporting information



Supporting Information

Supporting Information
