# Supplementary material for: Cognitive effects of dopaminergic treatment in Alzheimer's disease: Systematic review and meta‐analysis
Source: Alzheimers Dement (N Y). 2025 Aug 20;11(3):e70142. doi: 10.1002/trc2.70142 (PMC12365664; doi:10.1002/trc2.70142)
Supplement: Supplementary file 1 — Supporting Information [file TRC2-11-e70142-s002.docx]

**Supplementary Materials**

**Table S1. Checklist for meta-analysis of observational studies (noradrenergic meta-analysis)**

| Section/topic | # | Checklist item | Location Reported |
| --- | --- | --- | --- |
| TITLE | | |  |
| Title | 1 | Identify the report as a systematic review, meta-analysis, or both. | Title |
| ABSTRACT | | |  |
| Structured summary | 2 | Provide a structured summary including, as applicable: background; objectives; data sources; study eligibility criteria, participants, and interventions; study appraisal and synthesis methods; results; limitations; conclusions and implications of key findings; systematic review registration number. | Abstract |
| INTRODUCTION | | |  |
| Rationale | 3 | Describe the rationale for the review in the context of what is already known. | Introduction |
| Objectives | 4 | Provide an explicit statement of questions being addressed with reference to participants, interventions, comparisons, outcomes, and study design (PICOS). | Introduction |
| METHODS | | |  |
| Protocol and registration | 5 | Indicate if a review protocol exists, if and where it can be accessed (e.g. web address), and, if available, provide registration information including registration number. | N/A |
| Eligibility criteria | 6 | Specify study characteristics (e.g., PICOS, length of follow-up) and report characteristics (e.g., years considered, language, publication status) used as criteria for eligibility, giving rationale. | Methods, Table 1 |
| Information sources | 7 | Describe all information sources (e.g., databases with dates of coverage, contact with study authors to identify additional studies) in the search and date last searched. | Methods |
| Search | 8 | Present full electronic search strategy for at least one database, including any limits used, such that it could be repeated. | Supplementary Materials |
| Study selection | 9 | State the process for selecting studies (i.e., screening, eligibility, included in systematic review, and, if applicable, included in the meta-analysis). | Methods, Figure S1 |
| Data collection process | 10 | Describe method of data extraction from reports (e.g., piloted forms, independently, in duplicate) and any processes for obtaining and confirming data from investigators. | Methods |
| Data items | 11 | List and define all variables for which data were sought (e.g., PICOS, funding sources) and any assumptions and simplifications made. | Methods |
| Risk of bias in individual studies | 12 | Describe methods used for assessing risk of bias of individual studies (including specification of whether this was done at the study or outcome level), and how this information is to be used in any data synthesis. | Methods, Figure S2 |
| Summary measures | 13 | State the principal summary measures (e.g., risk ratio, difference in means). | Results, Figure 1 |
| Synthesis of results | 14 | Describe the methods of handling data and combining results of studies, if done, including measures of consistency (e.g., I^2^) for each meta-analysis. | Methods, Results, Figure 1 |

| **Inclusion Criteria** | **Exclusion Criteria** |
| --- | --- |
| Published between 1980-2023 | Studies prior to 1980 |
| Peer-reviewed | Single dose studies |
| Prospective trial | Not in English |
| Placebo controlled | No placebo group included |
| More than 1 participant | Conference abstracts |
| Any age range | Editorials, review articles, letters, or case reports |
| English language | Duplicate data |
| Looking exclusively at dopaminergic drugs | Poorly defined patient cohort; e.g. ‘dementia’ |
| Study includes predominantly patients with the included diagnoses | Non-accessible data |
| Study must report a change in recognized score of cognition |  |
| For studies reporting duplicate data, the most recent or most comprehensive publication to be indexed |  |

**Table S2.** Full inclusion and exclusion criteria for studies in this meta-analysis.

**Key terms:** Search was done using the following terms ($ is used as a truncation command): ((Alzheimer$ or mild cognitive impairment) and (cogniti$ or behav$ or psychiatric or psychological or apathy or memory or attention) and (dopamine* or stimulant or *amphetamine or methylphenidate or *modafinil or l-dopa or levodopa or bromocriptine or amantadine or cabergoline or quinagolide or lisuride or pergolide or apomorphine or ropinirole or pramipexole or piribedil or rotigotine or brexpiprazole or aripiprazole or cariprazine or bifeprunox or vesicular monoamine transporter inhibitor or catechol-O-methyltransferase or Tyrosine hydroxylase inhibitor or Aromatic L-amino acid decarboxylase inhibitor) and (trial or control$ or experimental or placebo).ab,mf,ot,ti,tn,dq)

**
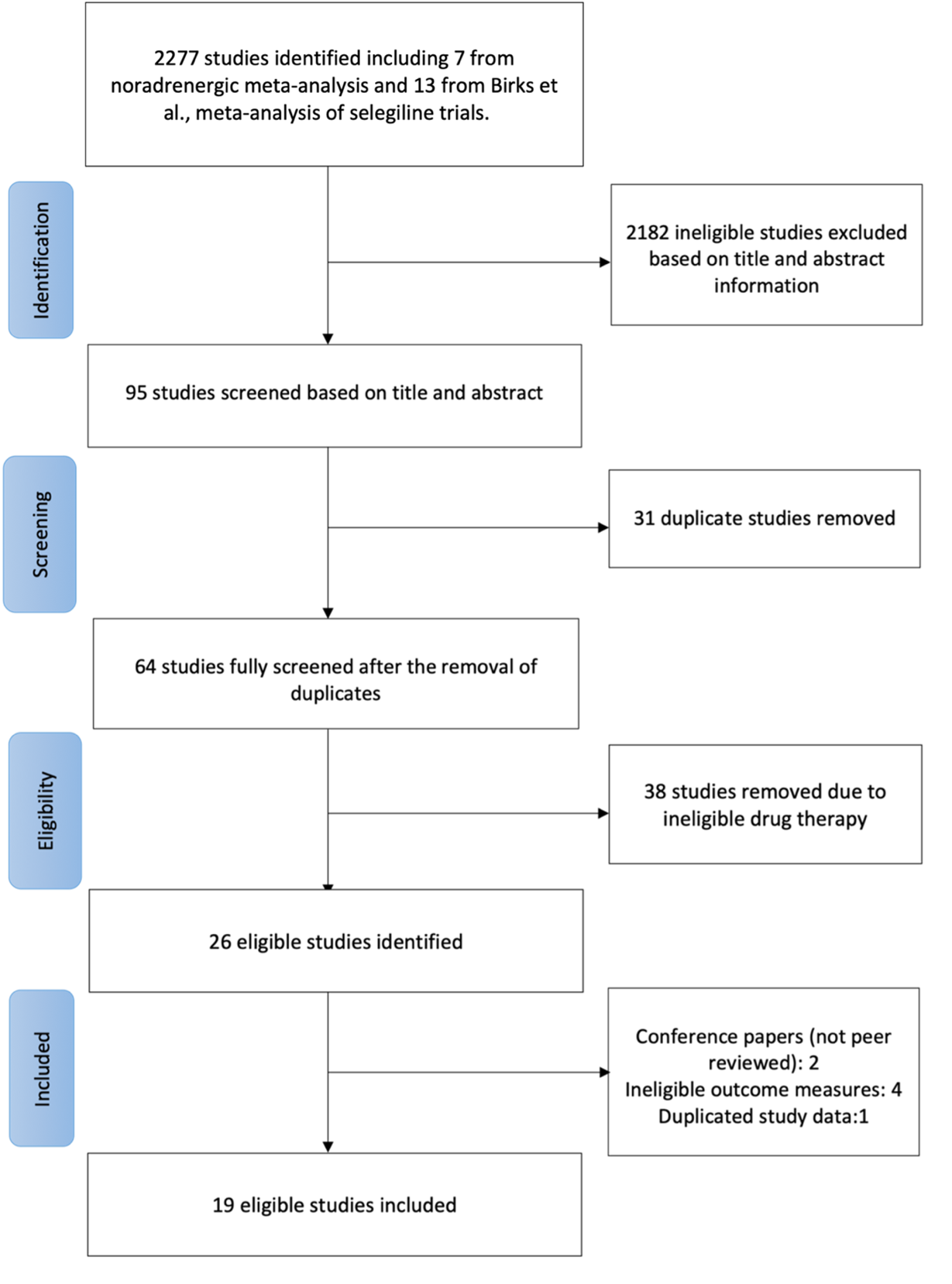
**

***Figure S1.*** *Preferred reporting items for Systematic Reviews and Meta-Analysis (PRISMA) flow diagram for search for studies reporting the use of dopaminergic therapies in AD. Some records excluded for more than one reason.*

| **Table S3. Quality assessment of dopaminergic studies using the NIH Quality Assessment Tool for Controlled Intervention Studies** | | | | | | | | | | | | | | | | |
| --- | --- | --- | --- | --- | --- | --- | --- | --- | --- | --- | --- | --- | --- | --- | --- | --- |
| Study | Patient group | Quality assessment question | | | | | | | | | | | | | | Quality rating |
|  |  | 1 | 2 | 3 | 4 | 5 | 6 | 7 | 8 | 9 | 10 | 11 | 12 | 13 | 14 |  |
| Herrmann2008 | AD | Y | NR | Y | Y | Y | NR | Y | Y | Y | Y | Y | N | Y | Y | Fair |
| Lanctôt2014 | AD | Y | Y | Y | Y | Y | Y | Y | Y | Y | Y | Y | Y | Y | Y | Good |
| Maier2020 | AD | Y | Y | Y | Y | Y | Y | N | Y | Y | Y | Y | N | Y | Y | Poor |
| Mintzer2021 | AD | Y | Y | Y | Y | Y | Y | Y | Y | Y | Y | Y | Y | Y | Y | Good |
| Padala2018 | AD | Y | Y | Y | Y | Y | Y | Y | Y | Y | Y | Y | N | Y | Y | Fair |
| Claus1998 | AD | Y | NR | Y | Y | Y | Y | Y | Y | Y | NR | Y | NR | Y | Y | Fair |
| Filip1999 | AD | Y | Y | Y | Y | Y | NR | Y | Y | Y | Y | Y | NR | Y | Y | Good |
| Koch2020 | AD | Y | Y | Y | Y | Y | Y | Y | N | Y | Y | Y | Y | Y | Y | Fair |
| Matthews2021 | AD | Y | Y | Y | Y | Y | Y | Y | Y | Y | Y | Y | Y | Y | Y | Good |
| Tariot1998 | AD | Y | CD | Y | Y | Y | N | Y | Y | Y | Y | Y | NR | Y | Y | Poor |
| Schneider2019 | AD | Y | Y | Y | Y | Y | Y | Y | Y | Y | Y | Y | Y | Y | Y | Good |
| Sano1996 | AD | Y | NR | Y | Y | Y | Y | Y | Y | Y | CD | Y | Y | Y | Y | Good |
| Mangoni1991 | AD | Y | NR | Y | Y | Y | Y | Y | Y | Y | Y | Y | N | Y | Y | Fair |
| Lawlor1997 | AD | Y | Y | Y | Y | Y | NR | Y | Y | Y | NR | Y | NR | Y | Y | Fair |
| Freedman1996 | AD | Y | Y | Y | Y | Y | Y | Y | Y | Y | Y | Y | Y | Y | Y | Good |
| Finali1991 | AD | Y | NR | Y | Y | Y | Y | Y | Y | Y | Y | Y | N | Y | Y | Fair |
| Burke1993 | AD | Y | NR | Y | Y | Y | CD | Y | Y | Y | NR | Y | NR | Y | Y | Poor |
| Agnoli1992 | AD | Y | NR | Y | Y | Y | Y | Y | Y | Y | Y | Y | N | Y | Y | Fair |
| Agnoli1990 | AD | N | NR | CD | Y | Y | Y | Y | Y | Y | Y | Y | NR | Y | Y | Poor |

AD = Alzheimer’s disease, MCI = mild cognitive impairment, PD = Parkinson’s disease, FTD = Frontotemporal dementia

Y = Yes; N = No; NA = not applicable; NR = not reported; CD = cannot determine.

Quality Rating: 1 in the ‘No’ column = Fair; >1 in the ‘No’ column = Poor. Consider Fair/Poor if too many questions cannot be answered.

Questions were:

1. Was the study described as randomized, a randomized trial, a randomized clinical trial, or an RCT?
2. Was the method of randomization adequate (i.e., use of randomly generated assignment)?
3. Was the treatment allocation concealed (so that assignments could not be predicted)?
4. Were study participants and providers blinded to treatment group assignment?
5. Were the people assessing the outcomes blinded to the participants' group assignments?
6. Were the groups similar at baseline on important characteristics that could affect outcomes (e.g., demographics, risk factors, co-morbid conditions)?
7. Was the overall drop-out rate from the study at endpoint 20% or lower of the number allocated to treatment?
8. Was the differential drop-out rate (between treatment groups) at endpoint 15 percentage points or lower?
9. Was there high adherence to the intervention protocols for each treatment group?
10. Were other interventions avoided or similar in the groups (e.g., similar background treatments)?
11. Were outcomes assessed using valid and reliable measures, implemented consistently across all study participants?
12. Did the authors report that the sample size was sufficiently large to be able to detect a difference in the main outcome between groups with at least 80% power?
13. Were outcomes reported or subgroups analyzed prespecified (i.e., identified before analyses were conducted)?
14. Were all randomized participants analyzed in the group to which they were originally assigned, i.e., did they use an intention-to-treat analysis?

**Figure S2. Funnel Plots of Dopaminergic Drugs in on Cognition in Alzheimer’s disease**


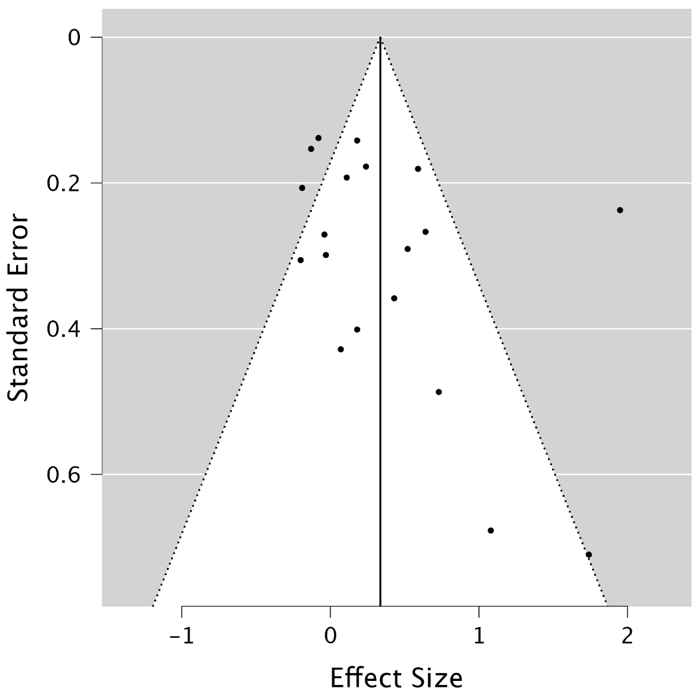


***Figure S2.*** *Funnel Plot to identify asymmetry that may be indicative of publication bias from dopaminergic meta-analysis on cognition*

| **Table S4.. Results of Meta-Regression on Dopaminergic Drugs: Global Cognition Analysis** | | | | |
| --- | --- | --- | --- | --- |
| Covariates | Number of studies | β (CI 95%) | P value | Proportion of variance explained |
| Age | 19 | -0.0265 (-0.0948, 0.0417) | 0.4237 | 0.00% |
| Gender (% female) | 19 | -0.0044 (-0.0224, 0.0103) | 0.4463 | 0.00% |
| Duration of treatment (weeks) | 19 | 0.0004 (-0.0086, 0.0094) | 0.9288 | 0.00% |
| Year of publication | 19 | -0.0233 (-0.0428, -0.0039) | 0.0214* | 28.05% |

**Figure S3. Bubble Plots for Meta-Regression of Effect of Covariates on Results of Dopaminergic Drugs on Cognition**


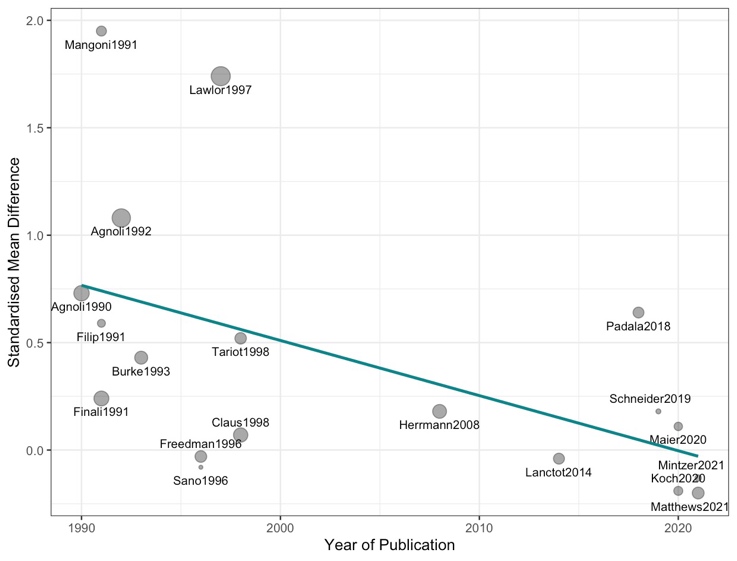

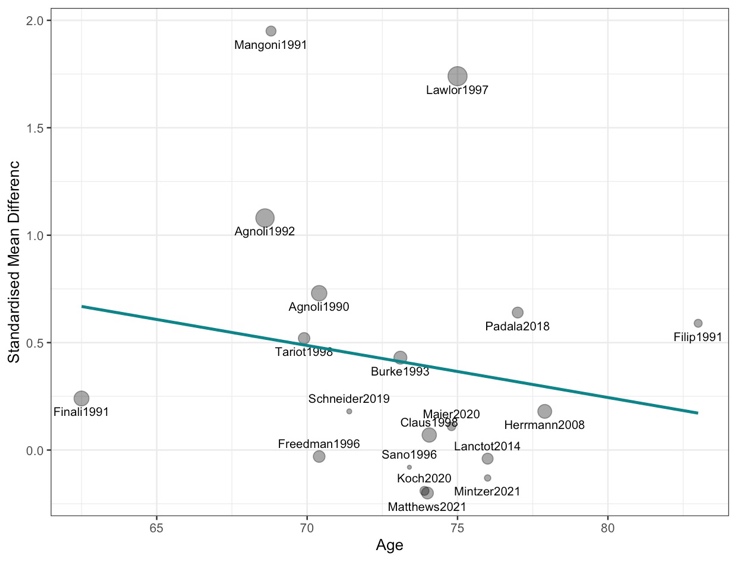

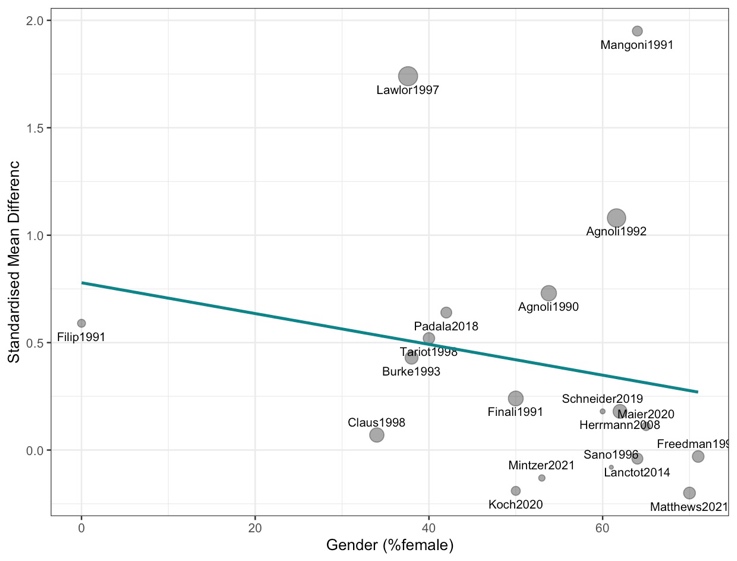

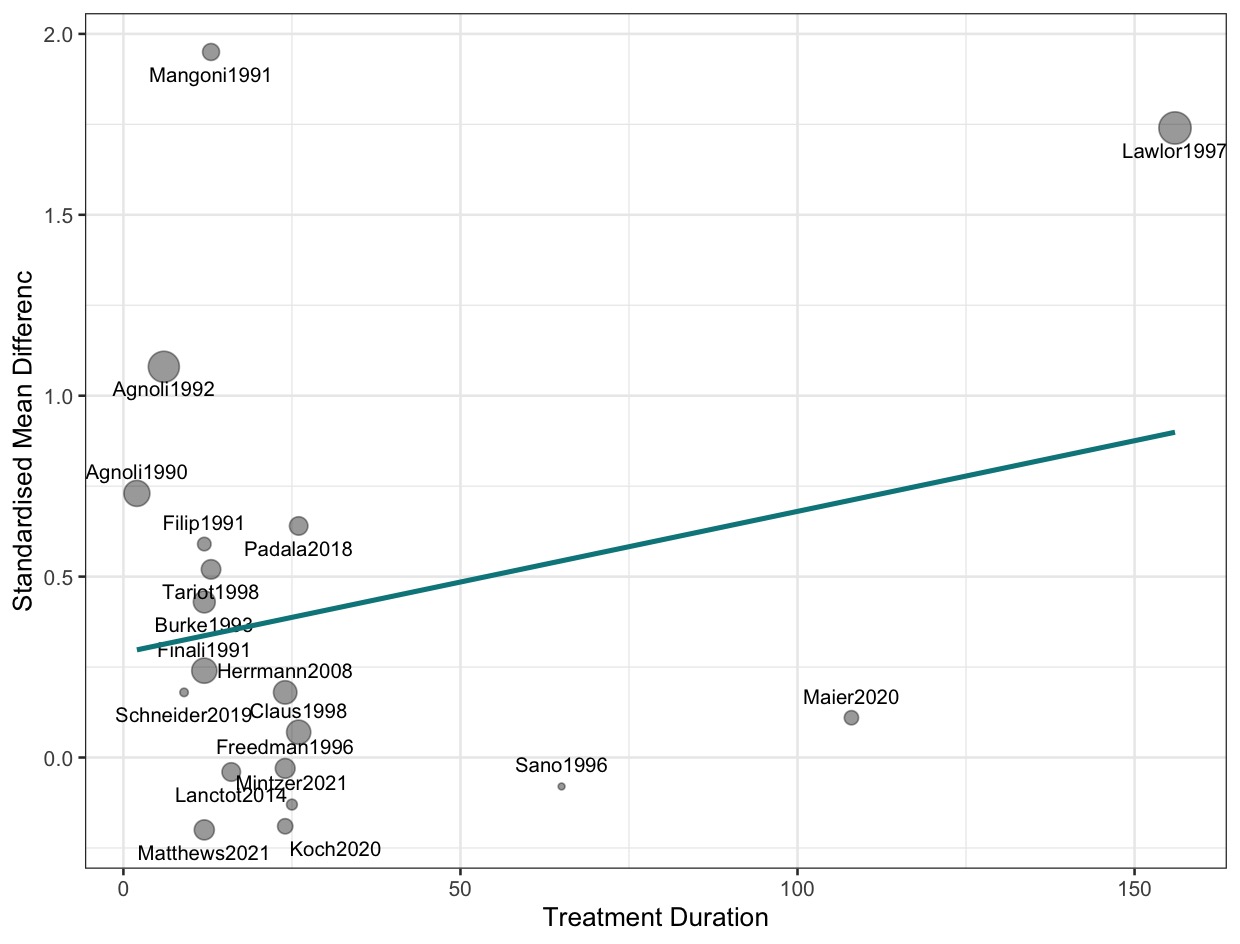


A)

B)

C)

D)

***Figure S3.*** *Bubble plots to investigate if covariates were significantly associated with effect size differences.* *Bubble size represents standard error. Year of publication the only significant relationship.*
